# Overexpression of the Mas1 gene mitigated LPS-induced inflammatory injury in mammary epithelial cells by inhibiting the NF-κB/MAPKs signaling pathways

**DOI:** 10.3389/fvets.2024.1446366

**Published:** 2024-07-12

**Authors:** Shuping Yan, Xianghong Ju, Jianlong Lao, Zhaohai Wen, Yanhong Yong, Yin Li, Youquan Li

**Affiliations:** ^1^Department of Veterinary Medicine, College of Coastal Agricultural Sciences, Guangdong Ocean University, Zhanjiang, China; ^2^Marine Medical Research and Development Centre, Shenzheng Institute of Guangdong Ocean University, Shenzheng, China

**Keywords:** EpH4 EV cells, Mas1, overexpression, inflammatory injury, blood-milk barrier

## Abstract

Breast infection is the primary etiology of mastitis in dairy cows, leading to a reduction in the quality of dairy products and resulting in substantial economic losses for animal husbandry. Although antibiotic treatment can eliminate the pathogenic microorganisms that induce mastitis, it cannot repair the inflammatory damage of mammary epithelial cells and blood milk barrier. Mas1 is a G protein-coupled receptor, and its role in lipopolysaccharide (LPS) -induced inflammatory injury to mammary epithelial cells has not been studied. LPS treatment of EpH4 EV cells led to a significant downregulation of Mas1 transcript levels, which attracted our great interest, suggesting that Mas1 may be an important target for the treatment of mastitis. Therefore, this study intends to verify the role of Mas1 in the inflammatory injury of EpH4 EV cells by gene overexpression technology and gene silencing technology. The findings demonstrated that the overexpression of the Mas1 gene effectively reversed the activation of the nuclear factor-κB/mitogen-activated protein kinase (NF-κB/MAPK) signaling pathways induced by LPS, while also suppressing the upregulation of pro-inflammatory mediators. Furthermore, overexpression of the Mas1 gene reversed the downregulation of zonula occludens 1 (ZO-1), Occludin, and Claudin-3 caused by LPS, suggesting that Mas1 could promote to repair the blood-milk barrier. However, the silencing of the Mas1 gene using siRNA resulted in a contrasting effect. These results indicated that Mas1 alleviated the inflammatory injury of mammary epithelial cells induced by LPS.

## 1 Introduction

Mastitis is a serious disease that threatens human and animal health, as well as leading to substantial financial losses on a global scale (1–3). Its main feature is the inflammatory injury of mammary epithelial cells (4). *Staphylococcus aureus*, *Escherichia coli*, and *Streptococcus* are the main pathogens causing cow mastitis (5–7). *Escherichia coli* is considered to be one of the most common pathogens of cow mastitis, and LPS is its main pathogenic factor, which can destroy the blood-milk barrier of cows (8). Antibiotic treatment is the main means to control cow mastitis. Although antibiotics can eliminate pathogenic bacteria, they cannot repair the barrier function damage caused by pathogens (9). Therefore, it is significant to strengthen the research on the pathogenic mechanism of mastitis and explore the key target molecules of immune prevention and control of mastitis in dairy cows.

An increasing body of research has substantiated the pivotal role of the renin-angiotensin system in exerting anti-inflammatory and anti-injury effects (10–12). Mas1 is a widely distributed G protein-coupled receptor expressed in various animal tissues (13). Mas1 plays a crucial role as one of the key components within the renin-angiotensin system, which is activated by angiotensin (1–7) to play anti-inflammatory, anti-fibrotic, and other roles (14, 15). The beneficial effects of Mas1 activation have been demonstrated in a variety of disease models (16–20). It is worth mentioning that our previous study proved that Angiotensin-converting enzyme 2 (ACE2; upstream of Mas1 receptor) plays an active therapeutic role in streptococcal mastitis (21). However, the role of Mas1 in mastitis remains to be elucidated.

In this investigation, LPS was employed to establish an inflammatory injury model of EpH4 EV cells to elucidate the advantageous function of Mas1 in ameliorating mastitis. This study clarified the role of Mas1 in mastitis, which could provide important targets for immunotherapy and drug development of cow mastitis.

## 2 Materials and methods

### 2.1 Modeling the injury of EpH4-Ev cells

EpH4 EV cells (mouse mammary epithelial cells) were kindly donated by Professor Yuanshu Zhang from Nanjing Agricultural University. Referring to previous studies (22), EpH4 EV cells were exposed to varying concentrations of LPS for a duration of 9 h to screen the optimal LPS concentration. According to the detection results of the CCK8 kit, the appropriate concentration of LPS was selected to treat EpH4 EV cells to construct the inflammatory injury model of mammary epithelial cells.

### 2.2 Construction of pVAX1-Mas1 overexpression plasmid

RNA extraction and cDNA synthesis refer to the study of Wen et al. (23). In brief, the Trizol method was utilized to extract total RNA from mouse kidneys, and the concentration of RNA was detected by nanodrop one ultra micro-UV spectrophotometer (Thermo Fisher Scientific, United States). The RNA was transcribed into complementary DNA (cDNA) with the help of a reverse transcription kit (SynScript^®^III RT SuperMix; Tsingke Biotech Co., Ltd., China). A pair of specific primers were designed based on the coding region sequence of the mouse Mas1 gene (GenBank: NM_008552.5) as documented on the NCBI website. The Mas1 gene of mice was amplified by using cDNA as a template and specific primers (Mas-F and Mas-R, Supplementary Table 1). PCR assays were conducted in a total reaction volume of 25 μL, consisting of 1 μL cDNA, 1 μL of each primer, 12.5 μL High Fidelity PCR Enzyme (2 × Primer Master Mix; Takara, Dalian, China), and 9.5 μL ddH_2_O. The amplification process was conducted according to the instructions provided in the kit and followed the protocol outlined by Wen et al. (24). The Mas1 gene was inserted into the pVAX1 eukaryotic expression vector by T4 ligase to construct the pVAX1-Mas1 recombinant plasmid, verified by enzyme digestion (*Hind* III and *Xho* I) and gene sequencing.

The eukaryotic expression plasmid was introduced into EpH4 EV cells by Lipofectamine transfection technology (Hieff Trans^®^ Liposomal Transfection Reagent, Yeasen, china), and the overexpression of the Mas1 gene in EpH4 EV cells was assessed using quantitative PCR and Western blot analysis.

### 2.3 Preparation of small interfering RNAs (siRNAs)

According to the mRNA sequence of mouse Mas1 as reported on the NCBI website, three specific siRNAs and one negative control siRNA were designed by using the online program of siRNA design.^1^ Different siRNAs were introduced into EpH4 EV cells with Lipofectamine RNAmax transfection reagent (Thermo Fisher Scientific, United States), and the transcription and expression of Mas1 gene in EpH4 EV cells were detected by qPCR and Western blot, and the siRNA with the best interference effect was selected for subsequent experiments.

### 2.4 Treatment of EpH4-Ev cells with pVAX1-Mas1 or siRNA was administered

Referring to previous studies (21), EpH4 EV cells were cultured in 6-well plates and pretreated with pVAX1-Mas1 and siRNA for 45 h when the cell density reached about 40 ~ 50%. After a 9-h treatment with LPS, the cells were subsequently subjected to sample collection for further analysis.

### 2.5 Detection of NAGase activity using commercially available assay kits

A commercially available N-acetyl-β-D-glucosaminidase (NAGase) activity assay kit (Jiancheng Bioengineering Institute, Nanjing, China) was used to measure the NAGase activity in the supernatant of EpH4 EV cells, and the NAGase activity level was quantified as U/L.

### 2.6 qRT-PCR assays

The relative transcript abundance of Mas1, IL-6, iNOS, and ZO-1 was analyzed by qPCR as previously described (25). The Trizol method was utilized to extract total RNA from EpH4 EV cells, and the concentration of RNA was detected by nanodrop one ultra micro-UV spectrophotometer (Thermo Fisher Scientific, United States). The RNA was transcribed into complementary DNA (cDNA) with the help of a reverse transcription kit (SynScript^®^III RT SuperMix; Tsingke Biotech Co., Ltd., China). Primer sequences were shown in Supplementary Table 2. The mouse β-actin gene was utilized as a reference gene in the present study. The qPCR run programs were: 95°C/30 s (1 cycle), 95°C/10 s (40 cycle), 60°C/30 s. The dissociation curve was produced using the following parameters: 95°C for 15 s, followed by 60°C for 60 s, and a final step of 95°C for 15 s. The data were analyzed based on the raw cycle thresholds (Ct) obtained using BIO-RAD CFX Connect software (BIO-RAD, United States) and employing the comparative delta–delta Ct (2 − ^ΔΔ Ct^) method.

### 2.7 Western blot assays

Cell samples were collected, and total protein was collected after cells were treated with cell lysate RIPA (Merck Sharp & Dohme Corp, United States). The BCA protein concentration assay kit was utilized to measure the protein sample concentrations in each group (Beyotime, China), and the protein samples were denatured by boiling in a metal bath. The 30 μg protein sample was separated using SDS-PAGE gel electrophoresis and then transferred to a PVDF membrane (Merck Sharp & Dohme Corp, United States) for additional analysis. The PVDF membranes were incubated with a 5% solution of skimmed milk powder at room temperature for 2 h to effectively block nonspecific binding sites. After the membranes were washed five times with Tris-buffered saline containing 0.1% Tween-20 (TBST), they were co-incubated with the primary antibody at 4°C overnight. The membranes were subjected to five washes with TBST and subsequently exposed to a secondary antibody for 2 h at room temperature. After the membranes were washed five times with TBST, they were transferred to the Tianneng chemiluminescence imager for luminescent imaging. The actin protein was utilized as an internal reference, and the relative expression abundance of the target protein was quantified using Image J software for analysis.

Source antibodies: Mas1 (Novus Biologicals, United States); TNF-α and IL-Iβ (Santa cruz, USA); IL-6, iNOS and ZO-1 (Affinity Biosciences, United States); p-p38(Thr180/Tyr182) and p38 (Cell Signaling Technology, United States); ERK, p-ERK(Thr202/Tyr204), p-p65 (phospho Ser536), p65, p-JNK (phospho Thr183/Y185), JNK, Occludin and Claudin-3 (Proteintech Group, Wuhan, China).

### 2.8 Statistical analysis

The results were reported as the mean ± SEM (standard error of the mean). The comparison between the LPS treatment group and the control group was analyzed using an independent-sample t-test with Compare Means by SPSS 21.0 (StatSoft, Inc., Tulsa, United States). The LPS treatment group was compared with other groups using one-way ANOVA. The results were presented as ^*^p < 0.05, ^**^p < 0.01 compared to the control group; ^#^*p* < 0.05 compared to the LPS group. The experiments were conducted with a minimum of three independent repetitions.

## 3 Results

### 3.1 The transcript level of the Mas1 gene was negatively correlated with the damage of EpH4 EV cells induced by LPS

**Figure 1:**
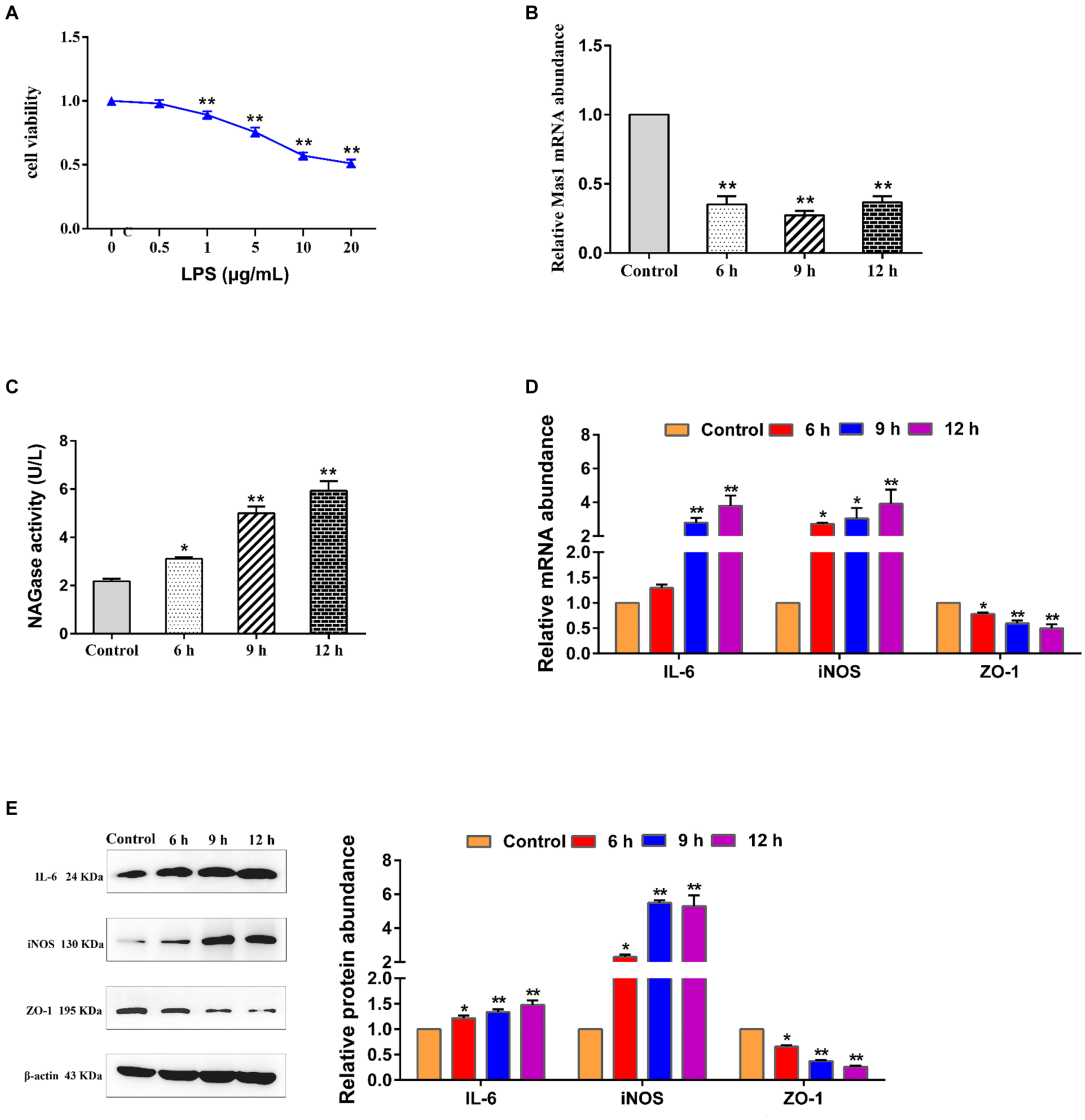
The transcript level of the Mas1 gene was negatively correlated with the damage of EpH4 EV cells induced by LPS. **(A)** EpH4 EV cells were exposed to varying concentrations of LPS for a duration of 9 h, and the activity of cells was detected by CCK8 kit. **(B)** EpH4 EV cells were treated with 5 μg/mL of LPS for varying durations, as per the experimental design, and the relative transcript abundance of the Mas1 gene was analyzed by qPCR. **(C)** EpH4 EV cells were treated with 5 μg/mL of LPS for varying durations, as per the experimental design, and the cell supernatant was assessed for NAGase activity by using a commercially available assay kit. **(D)** EpH4 EV cells were treated with 5 μg/mL of LPS for varying durations, as per the experimental design, and the relative transcript abundance of IL-6, ZO-1, and iNOS was analyzed by qPCR. **(E)** EpH4 EV cells were treated with 5 μg/mL of LPS for varying durations, as per the experimental design, and the relative expression abundance of IL-6, ZO-1, and iNOS proteins were analyzed by Western blot. The significance of differences was analyzed, with *indicating *p* < 0.05 and **indicating *p* < 0.01.

The effect t of varying concentrations of LPS on the activity of EpH4 EV cells was analyzed by using the CCK8 assay kit, and the optimal concentration of LPS was selected as 5 μg/mL (Figure 1A). Interestingly, the treatment of EpH4 EV cells with LPS resulted in a significant downregulation of the transcript abundance of the Mas1 gene, with the most pronounced effect observed at 9 h (Figure 1B). As we all know, NAGase is a marker to judge the damage of mammary epithelial cells (26, 27). Therefore, we analyzed the impact of LPS treatment on EpH4 EV cells by quantifying the activity of NAGase in a cell culture medium using a commercially available kit. The results showed that the activity of NAGase in the culture medium of EpH4 EV cells was significantly increased following LPS treatment (Figure 1C), indicating that the LPS-induced mammary epithelial cell model was successfully constructed. Moreover, qPCR and Western blot analysis revealed a significant upregulation of pro-inflammatory mediators (IL-6 and iNOS) as well as tight junction protein (ZO-1) transcription and expression in EpH4 EV cells following LPS treatment (Figures 1D,E). The above results show that the inflammatory injury of mammary epithelial cells induced by LPS was negatively correlated with the expression of Mas1 gene, suggesting that Mas1 gene may be a target molecule to improve mastitis.

### 3.2 Construction and validation of overexpression plasmid pVAX1-Mas1

**Figure 2:**
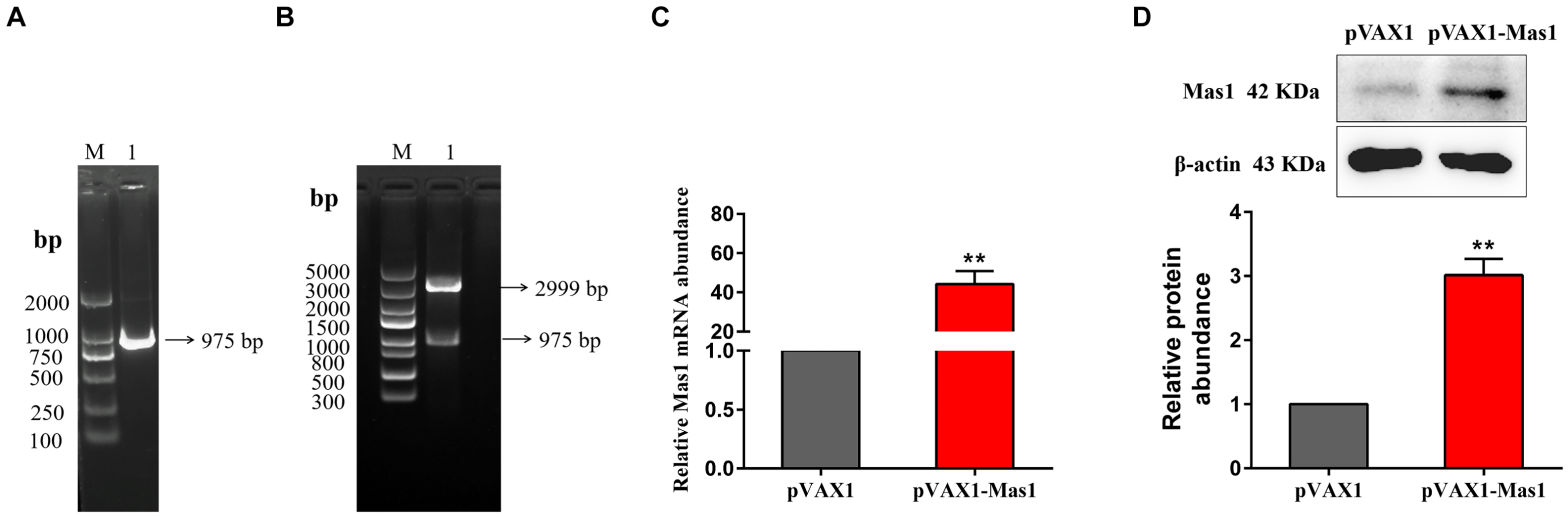
Construction and validation of overexpression plasmid pVAX1-Mas1. **(A)**, The amplification of Mas1 gene. Lane M displays the DNA Marker DL 2000, while Lane 1 displays the amplification products of the Mas1 gene. **(B)** Lane M displays DNA Marker DL 5000; Lane 1: digestion of pVAX1-Mas1 by enzymes. **(C)** The relative transcript abundance of Mas1 was detected by qPCR. **(D)** The relative expression level of Mas1 was analyzed by Western blot. The significance of differences was analyzed, with **indicating *p* < 0.01.

The Mouse Mas1 gene was amplified by specific primers using mouse kidney cDNA as a template. Agarose gel showed that a single band appeared at about 975 bp (Figure 2A), suggesting that the target gene was successfully amplified. The Mas1 gene was effectively integrated into the pVAX1 eukaryotic expression vector by using T4 DNA ligase, and the pVAX1-Mas1 recombinant plasmid was successfully constructed by enzyme digestion verification (Figure 2B) and gene sequencing verification. The overexpression of pVAX1-Mas1 recombinant plasmid in EpH4 EV cells was verified by qPCR and Western blot. The transcript abundance of the Mas1 gene in the pVAX1-Mas1 group was significantly increased compared to the pVAX1 control group (Figure 2C). Western blot results showed that Mas1 was significantly upregulated in EpH4 EV cells after pVAX1-Mas1 recombinant plasmid transfection, which was consistent with the qPCR results (Figure 2D). These results indicate that Mas1 was successfully overexpressed in EpH4 EV cells.

### 3.3 Evaluation of the silencing effect of the Mas1 gene in EpH4 EV cells by siRNA

**Figure 3:**
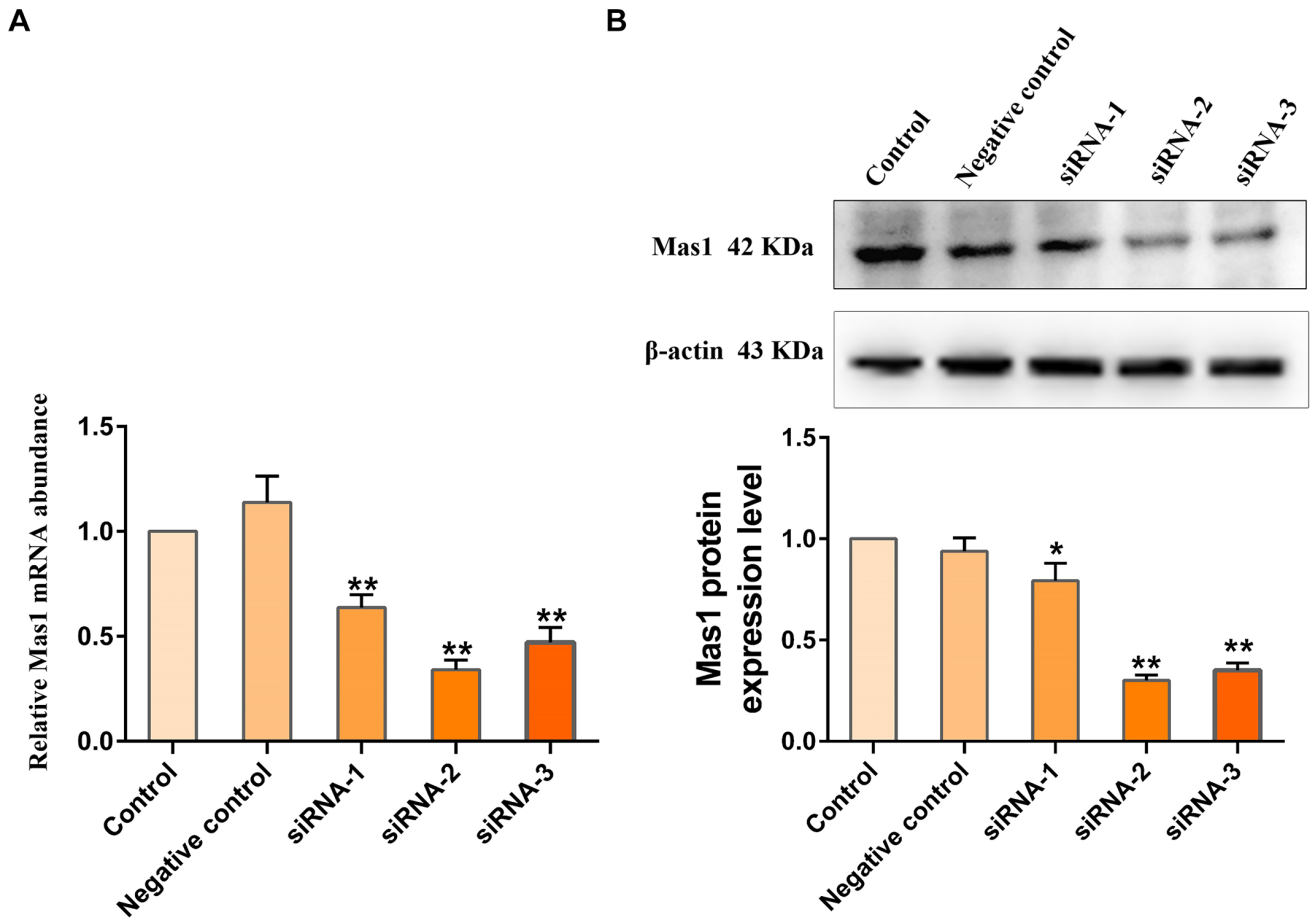
Evaluation of the silencing effect of Mas1 gene in EpH4 EV cells by siRNA. EpH4 EV cells were seeded in 6-well cell culture plates and treated with siRNA for 45 h when the cell density reached about 40–50%. **(A)** The relative transcript abundance of Mas1 was analyzed by qPCR. **(B)** The relative expression level of Mas1 was analyzed by Western blot. The significance of differences was analyzed, with **indicating *p* < 0.01.

To screen the siRNA with the best silencing effect on the Mas1 gene, the effects of different siRNA treatment groups on the transcription and expression levels of the Mas1 gene were detected through qPCR and Western blot assays. qPCR results showed that compared with the control group, the transcript abundance of Mas1 gene in siRNA-1, siRNA-2, and siRNA-3 treatment groups was significantly downregulated (Figure 3A), and the silencing effect of siRNA-2 group was the best. The results of the Western blot were consistent with those of qPCR. The expression levels of Mas1 protein in siRNA-1, siRNA-2, and siRNA-3 treatment groups were significantly downregulated (Figure 3B), and the effect of the siRNA-2 group was the best.

### 3.4 Effect of overexpression or silencing of Mas1 gene on NAGase activity

**Figure 4:**
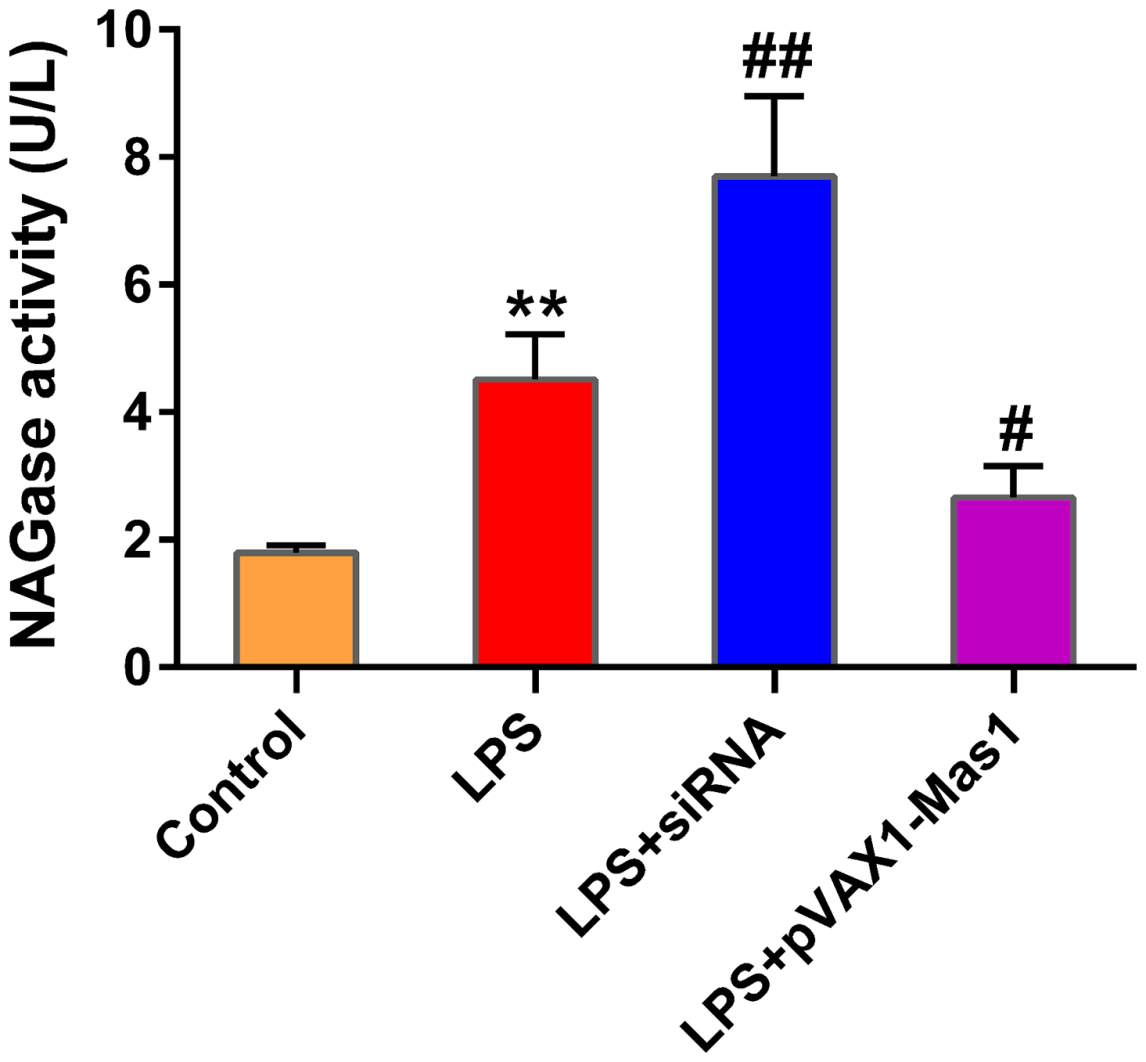
Effect of overexpression or silencing of Mas1 gene on NAGase activity. The cell supernatant was assessed for NAGase activity by using a commercially available assay kit. Significance is indicated by * and #, ^*^p < 0.05 compared to the control group; ^#^*p* < 0.05 compared to the LPS group.

The overexpression of the Mas1 gene notably attenuated the upregulation of NAGase activity induced by LPS, which is an intriguing finding in our study (Figure 4). However, the silencing of the Mas1 gene yields contrasting effects, further aggravating the increase of NAGase activity caused by LPS (Figure 4).

### 3.5 Effect of overexpression or silencing of Mas1 gene on the expression level of inflammatory mediators

**Figure 5:**
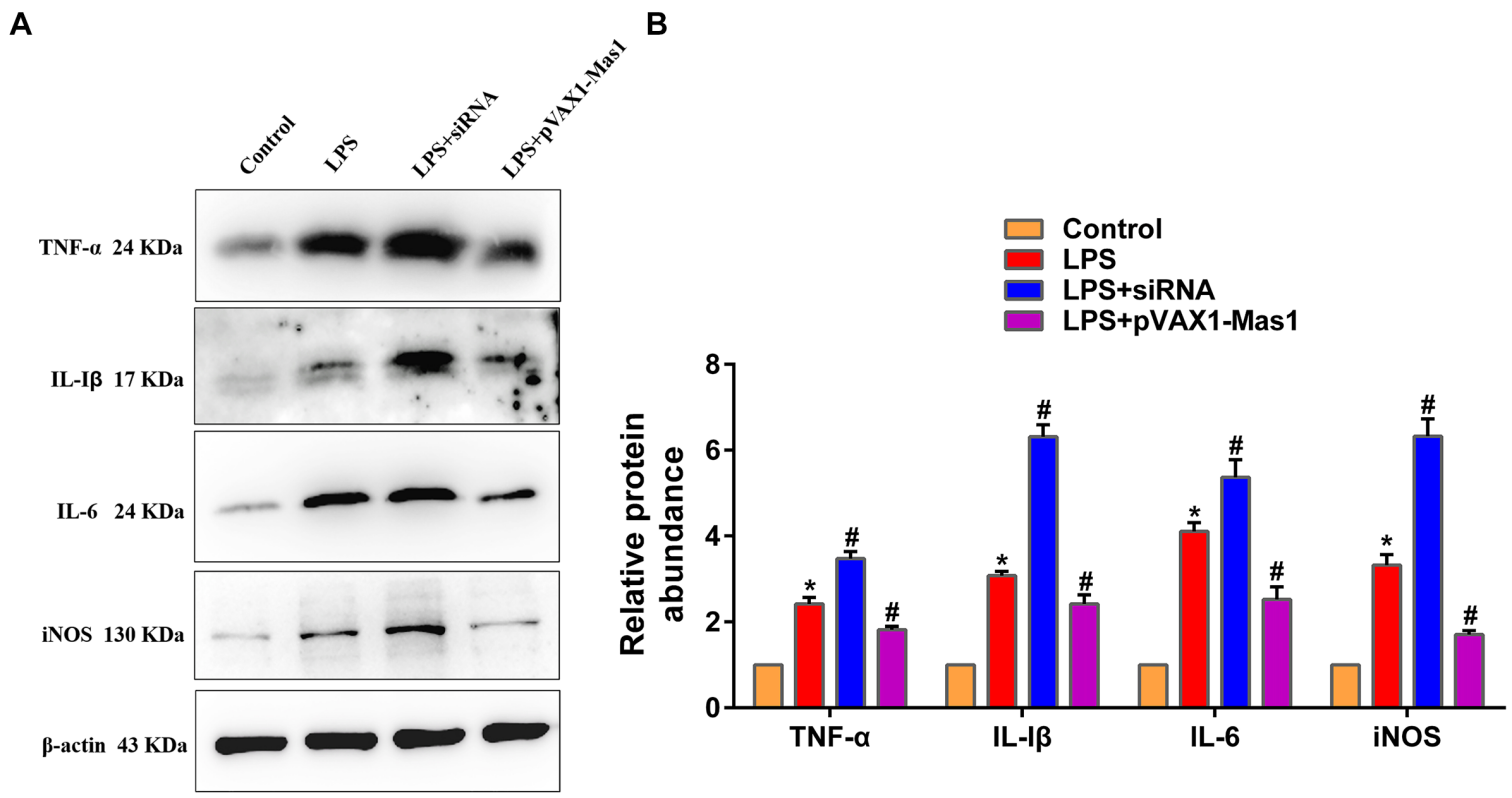
Effect of overexpression or silencing of Mas1 gene on the expression level of inflammatory mediators. **(A)** Quantification of the relative protein expression levels of IL-Iβ, IL-6, TNF-α, and iNOS using Western blot analysis. **(B)**, Western blot results for IL-Iβ, IL-6, TNF-α, and iNOS were analyzed to obtain statistical data. Significance is indicated by * and #, ^*^p < 0.05 compared to the control group; ^#^*p* < 0.05 compared to the LPS group.

Results as shown in Figures 5A,B, overexpression of the Mas1 gene led to a significant reversal of the upregulation of inflammatory mediators (iNOS, IL-Iβ, IL-6, and TNF-α) induced by LPS. However, the silencing of the Mas1 gene yields contrasting effects, further exacerbating the increased expression level of inflammatory mediators (iNOS, IL-Iβ, IL-6, and TNF-α) caused by LPS.

### 3.6 Effect of overexpression or silencing of Mas1 gene on NF-κB signaling pathway

**Figure 6:**
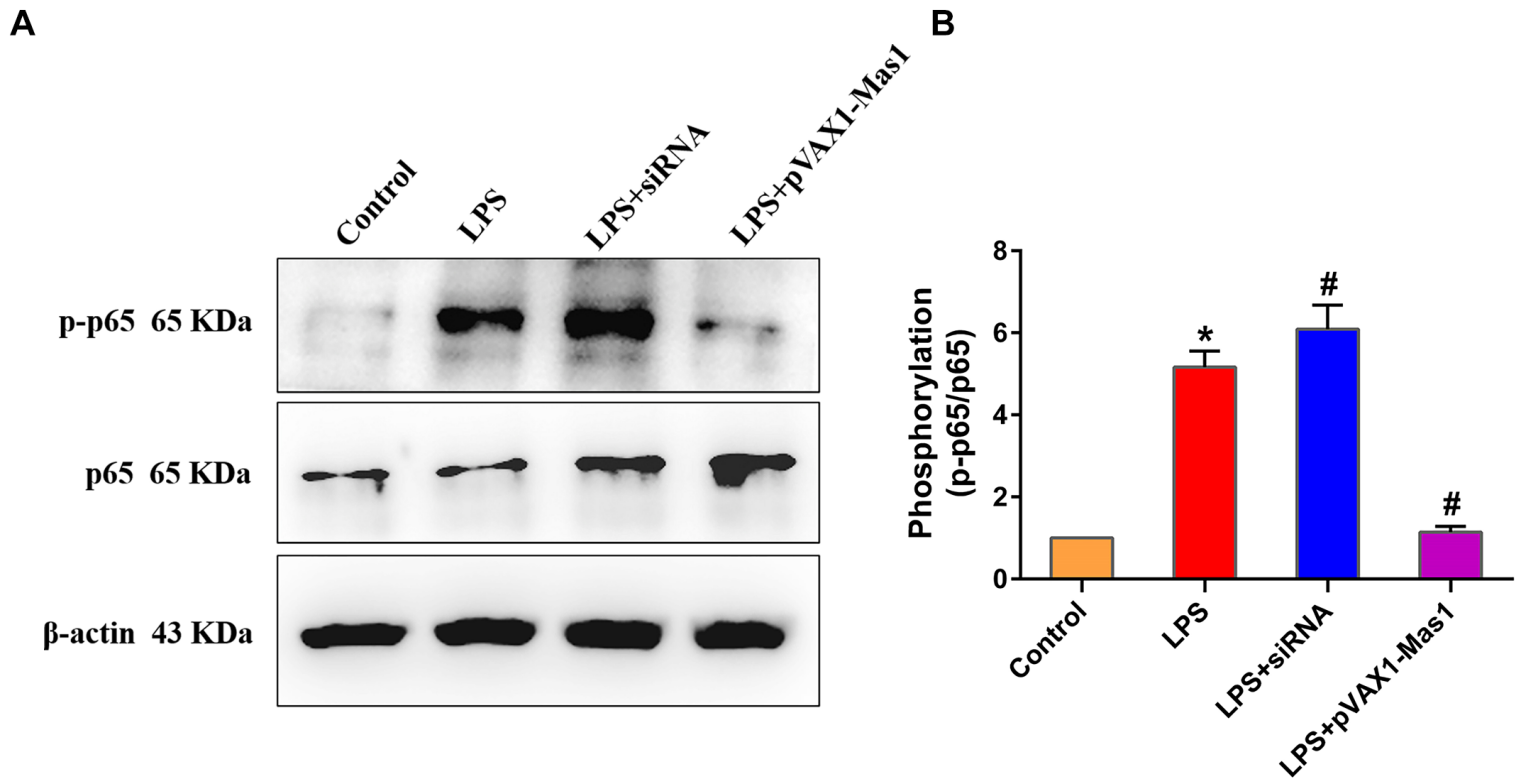
Effect of overexpression or silencing of Mas1 gene on NF-κB signaling pathway. **(A)** Quantification of the relative protein expression levels of p-p65 and p65 using Western blot analysis. **(B)** Western blot results for p-p65 and p65 were analyzed to obtain statistical data. Significance is indicated by * and #, ^*^p < 0.05 compared to the control group; ^#^*p* < 0.05 compared to the LPS group.

The phosphorylation level of p65 was significantly up-regulated in the LPS treated group compared to the control, indicating that LPS activated the NF-κB signaling pathway (Figures 6A,B). Interestingly, overexpression of the Mas1 gene significantly reversed the up-regulation of the p56 phosphorylation level induced by LPS (Figures 6A,B). On the contrary, silencing the Mas1 gene further aggravated the increase of the p56 phosphorylation level caused by LPS (Figures 6A,B).

### 3.7 Effect of overexpression or silencing of Mas1 gene on MAPKs signaling pathway

**Figure 7:**
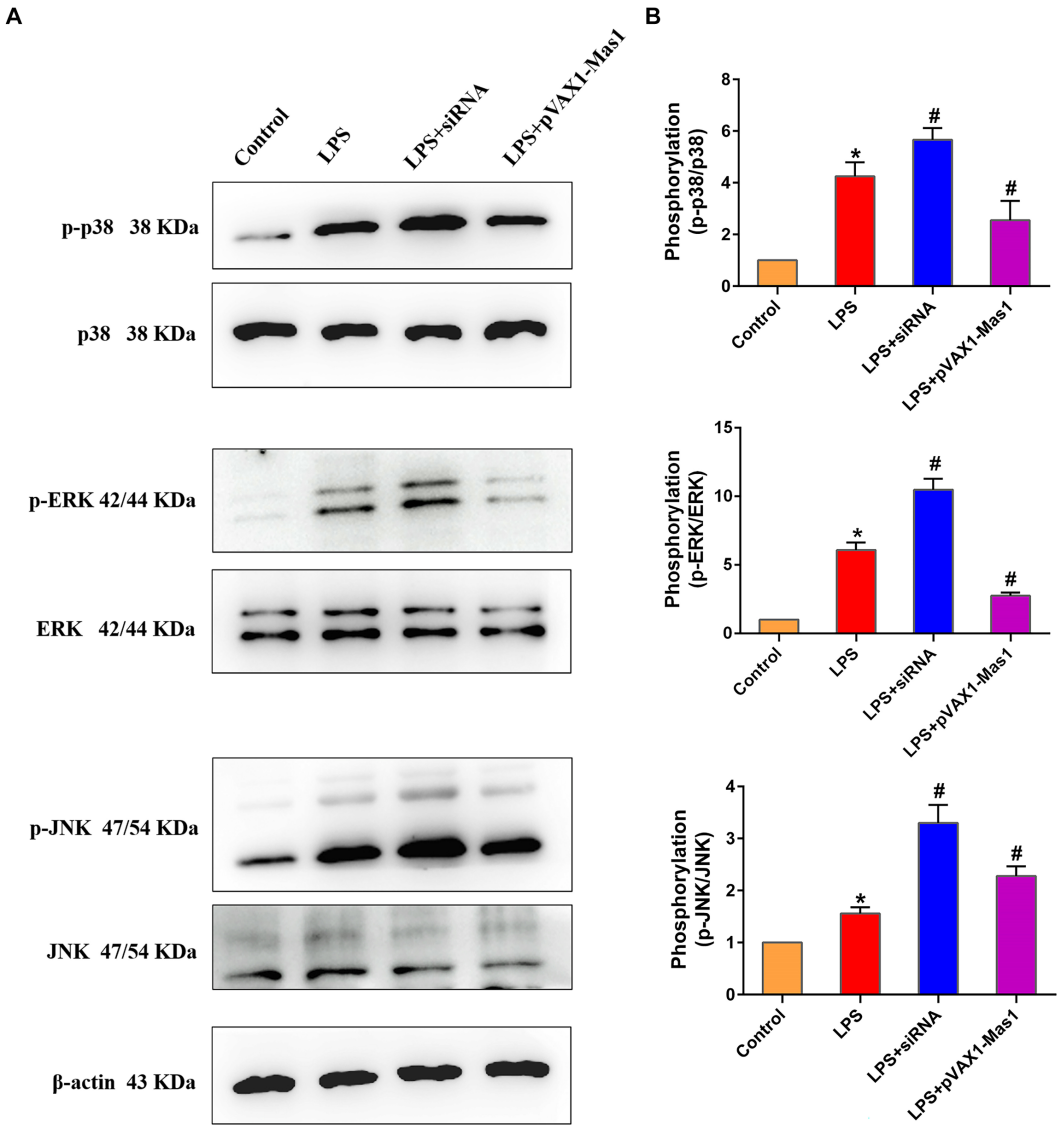
Effect of overexpression or silencing of Mas1 gene on MAPKs signaling pathway. **(A)** Quantification of the relative protein expression levels of ERK, p38, JNK, as well as phosphorylated forms (p-ERK, p-p38, p-JNK) using Western blot analysis. **(B)** Western blot results for ERK, p38, JNK, as well as phosphorylated forms of ERK, p38, and JNK were analyzed to obtain statistical data. Significance is indicated by * and #, ^*^p < 0.05 compared to the control group; ^#^*p* < 0.05 compared to the LPS group.

The phosphorylation level of p38, JNK, and ERK proteins were significantly up-regulated in LPS treated group compared to the control, indicating that LPS activated the MAPKs signaling pathway (Figure 7). Interestingly, overexpression of the Mas1 gene significantly reversed the up-regulation of the p-p38, p-JNK, and p-ERK protein levels induced by LPS (Figure 7). On the contrary, silencing Mas1 gene further aggravated the upregulation of p-p38, p-JNK, and p-ERK protein levels caused by LPS.

### 3.8 Effect of overexpression or silencing of Mas1 gene on the expression level of key proteins of blood milk barrier

**Figure 8:**
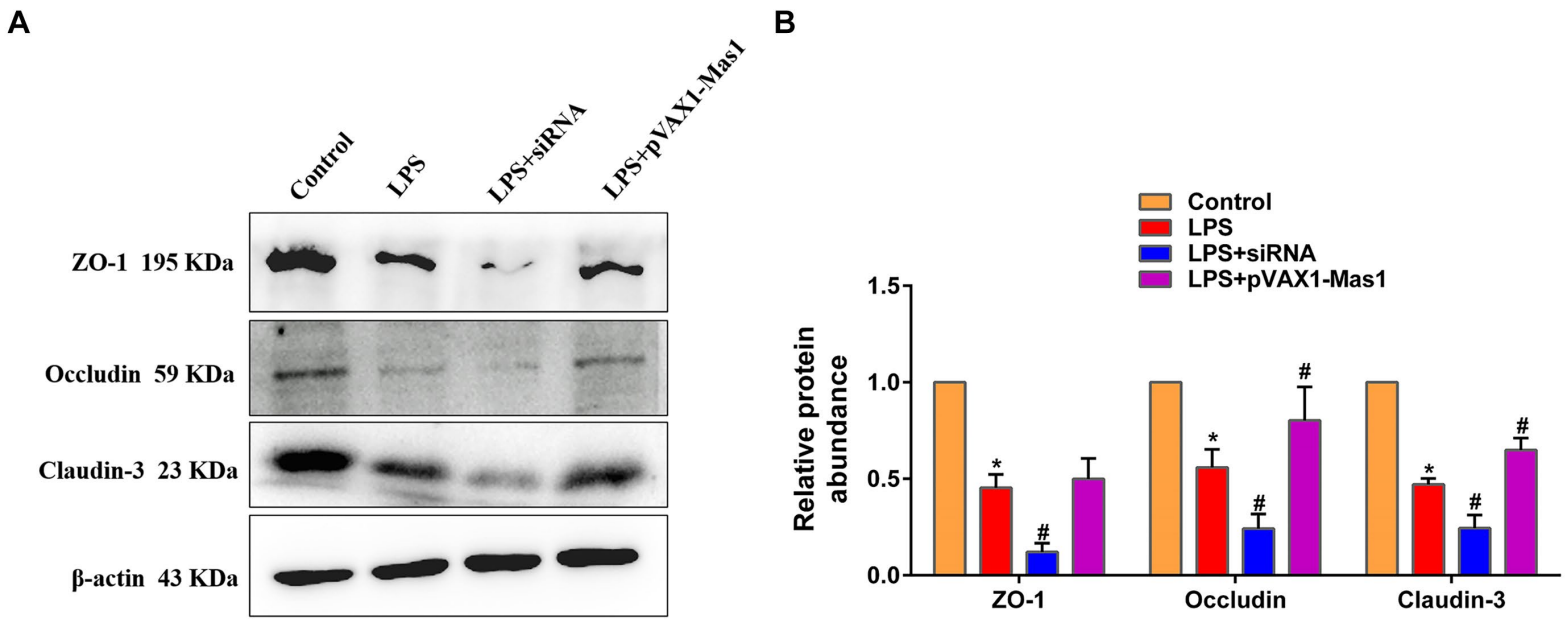
Effect of overexpression or silencing of Mas1 gene on the expression level of key proteins of blood milk barrier. **(A)** Quantification of the relative protein expression levels of Occludin, ZO-1, and Claudin-3 using Western blot analysis. **(B)** Western blot results for Occludin, ZO-1, and Claudin-3 were analyzed to obtain statistical data. Significance is indicated by * and #, ^*^p < 0.05 compared to the control group; ^#^*p* < 0.05 compared to the LPS group.

The expression abundance of key proteins (ZO-1, Claudin-3, and Occludin) of the blood-milk barrier was significantly downregulated in the LPS treatment group compared to the control group (Figure 8). Interestingly, overexpression of the Mas1 gene significantly reversed the down-regulated of Occludin and Claudin-3 protein abundance induced by LPS (Figure 8). On the contrary, silencing Mas1 gene further aggravated the down-regulation of tight junction protein (ZO-1, Claudin-3, and Occludin) expression caused by LPS.

## 4 Discussion

The mammary epithelial cells serve as the primary cellular component for the mammary gland and play a crucial role in the biosynthesis and excretion of milk fat and milk protein (8). In cow mastitis, the primary targets of pathogen attack are the mammary epithelial cells. As we all know, LPS is the main virulence factor of bacteria and is widely used to induce mastitis models (28, 29). In this study, following LPS treatment of EpH4 EV cells at different times, a significant upregulate in NAGase activity was observed in the cell supernatant. Additionally, there was a notable upregulation in the expression levels of inflammatory mediators within the cells, indicating that LPS treatment induced inflammatory injury to mammary epithelial cells. It is worth noting that after LPS treatment of EpH4 EV cells at different times, the transcription level of the Mas1 gene was significantly downregulated, which was negatively correlated with the inflammatory injury to mammary epithelial cells, suggesting that LPS may induce mastitis by downregulating the expression of Mas1 gene.

The damage to mammary epithelial cells will release NAGase into the milk, so it has also become the main biomarker for the clinical diagnosis of cow mastitis (26, 30). Our previous investigation demonstrated that the increased expression of the ACE2 gene effectively counteracted the elevation of NAGase activity induced by Streptococcus, providing evidence for the beneficial role of ACE2 in mastitis (21).

Previous literature has confirmed that the renin-angiotensin system includes two axes, the ACE2/Ang-(1–7)/Mas1 and the ACE/Ang-II/AT1 axis (31, 32). Mas1 is the downstream receptor of ACE2/Ang-(1–7)/Mas1, so the beneficial effect of overexpressing the ACE2 gene in mastitis may be the result of activating the Mas1 receptor. In line with anticipated outcomes, the present study observed a substantial reversal of LPS-induced elevation in NAGase activity following overexpression of the Mas1 gene, while silencing the Mas1 gene aggravated the LPS-induced increase in NAGase activity, indicating that Mas1 plays a beneficial role in LPS-induced breast epithelial cell injury.

The NF-κB\MAPKs signaling pathways are well-established inflammatory signaling cascades, whose activation can induce the production of downstream inflammatory mediators (such as iNOS, IL-Iβ, IL-6, and TNF-α) and initiate the inflammatory response (33–35). Therefore, key protein molecules in NF-κB and MAPKs signaling pathways have also become target molecules for anti-inflammatory drug development and screening (36–38). In this study, we observed that the overexpression of the Mas1 gene led to a significant reversal of LPS-induced activation of NF-κB/MAPKs signaling pathways, thereby mitigating inflammatory injury in EpH4 EV cells. However, the silencing of the Mas1 gene yields a contrasting effect. The findings align with the fluctuations in levels of inflammatory mediators. These results suggest that the Mas1 gene may be an important target molecule for immunotherapy and drug development of bovine mastitis.

The preservation of the integrity of the blood-milk barrier is crucial for the optimal functioning of lactation in mammary tissue (39, 40). More and more studies have confirmed that ZO-1, occludin, and Claudin-3 are key structural molecules that maintain the blood-milk barrier and play an important role in the process of resisting pathogen invasion (41–43). In this study, we observed that the overexpression of the Mas1 gene significantly reversed the downregulation of tight junction protein expression induced by LPS, which indicated that the overexpression of Mas1 repaired the damage of the blood milk barrier caused by LPS. However, the silencing of the Mas1 gene yields a contrasting effect. These results suggest that upregulating the expression of key proteins of the blood-milk barrier may be one of the ways for the Mas1 gene to alleviate mastitis.

In this study, we demonstrated the role of the Mas1 gene in inflammatory injury of mammary epithelial cells by overexpression and RNA interference technology. However, these findings are limited to *in vitro* studies, and our future research will focus on conducting *in vivo* studies to further validate the role of the Mas1 gene in various mastitis models.

## 5 Conclusion

This study demonstrated that overexpression of the Mas1 gene effectively reversed the LPS-induced activation of NF-κB/MAPKs signaling pathways and mitigated the inflammatory damage to mammary epithelial cells. Moreover, overexpression of the Mas1 gene promoted the expression of tight junction proteins. These findings imply that the Mas1 gene could serve as a crucial focus for immunotherapeutic interventions in mastitis.

## Data availability statement

The original contributions presented in the study are included in the article/Supplementary material, further inquiries can be directed to the corresponding author/s.

## Ethics statement

The animal study was approved by Animal Ethics Committee of Guangdong Ocean University. The study was conducted in accordance with the local legislation and institutional requirements.

## Author contributions

SY: Conceptualization, Data curation, Validation, Writing – original draft, Writing – review & editing. XJ: Formal analysis, Supervision, Visualization, Writing – review & editing. JL: Investigation, Supervision, Writing – review & editing. ZW: Formal analysis, Methodology, Software, Supervision, Writing – review & editing. YY: Software, Supervision, Writing – review & editing. YiL: Methodology, Supervision, Writing – review & editing. YoL: Data curation, Funding acquisition, Project administration, Resources, Supervision, Writing – review & editing.

## Footnotes

^1^ https://rnaidesigner.thermofisher.com
