# Supplementary material for: Overexpression of the Mas1 gene mitigated LPS-induced inflammatory injury in mammary epithelial cells by inhibiting the NF-κB/MAPKs signaling pathways
Source: Front Vet Sci. 2024 Jul 12;11:1446366. doi: 10.3389/fvets.2024.1446366 (PMC11274334; doi:10.3389/fvets.2024.1446366)
Supplement: Supplementary file 1 [file Table_1.DOCX]

Supplementary Material

# Supplementary Tables

**Table 1. Primers used for PCR Amplification experiments**

| **Target genes** | **Primer sequences (5’-3’)** |
| --- | --- |
| Mas1 | CGGGATCCCCACCATGGACCAGTCAAATATGACATCC |
|  | CCGCTCGAGTCAGACCACAGTCTCAATGGAT |

Note: The underlined sequences are the *Hind* III and *Xho* I digestion sites.

**Table 2. Primers used for qPCR experiments**

| **Target genes** | **Primer sequences (5’-3’)** |
| --- | --- |
| β-actin | TCTGGCACCACACCTTCTA |
|  | AGGCATACAGGGACAGCAC |
| Mas1 | TTTCTATTTGGCTACAACACGGG |
|  | GGTAGAGGACCGATAGGCAC |
| IL-6 | CAAGAAAGACAAAGCCAGAGTC |
|  | GAAATTGGGGTAGGAAGGAC |
| iNOS | CACAGCAATATAGGCTCATCCA |
|  | GGATTTCAGCCTCATGGTAAAC |
| ZO-1 | GGGAGGGTCAAATGAAGACA |
|  | GGCATTCCTGCTGGTTACAT |
