# Supplementary figures and images for: Overexpression of the Mas1 gene mitigated LPS-induced inflammatory injury in mammary epithelial cells by inhibiting the NF-κB/MAPKs signaling pathways
Source: Front Vet Sci. 2024 Jul 12;11:1446366. doi: 10.3389/fvets.2024.1446366 (PMC11274334; doi:10.3389/fvets.2024.1446366)

**Figure 1E**

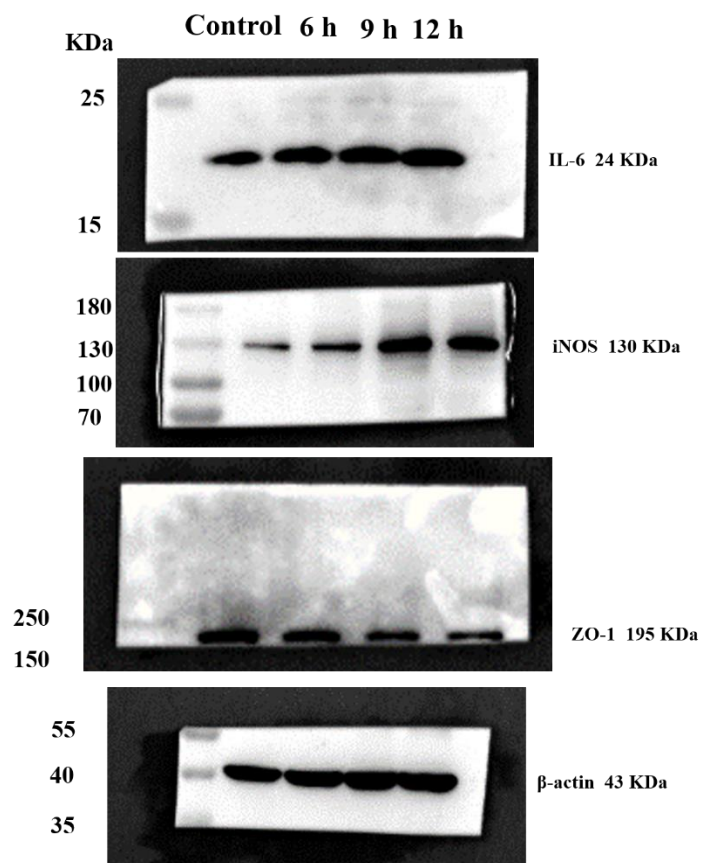

**Figure 2D**

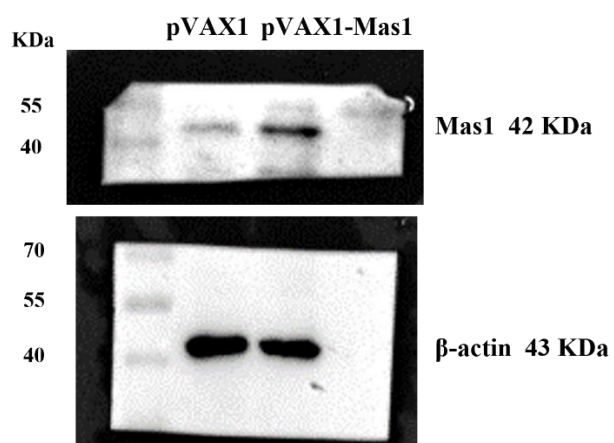

**Figure 3B**

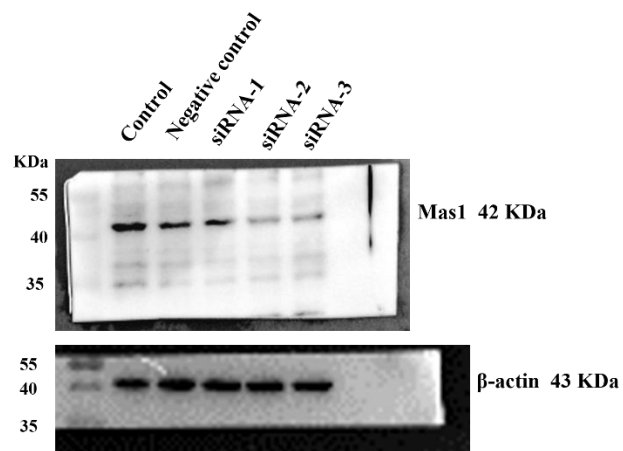

**Figure 5A**

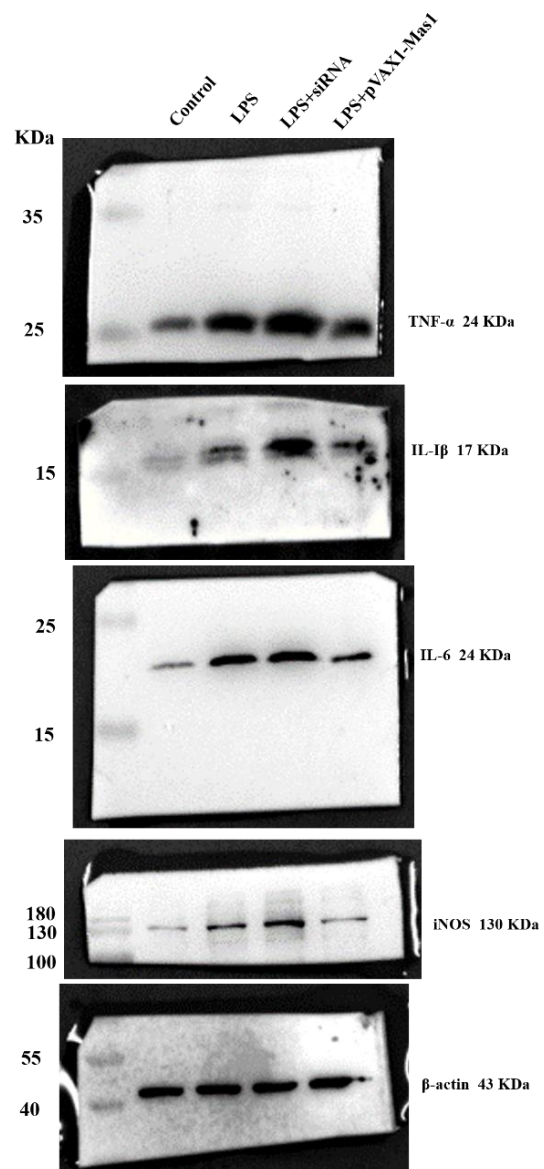

Figure 6A

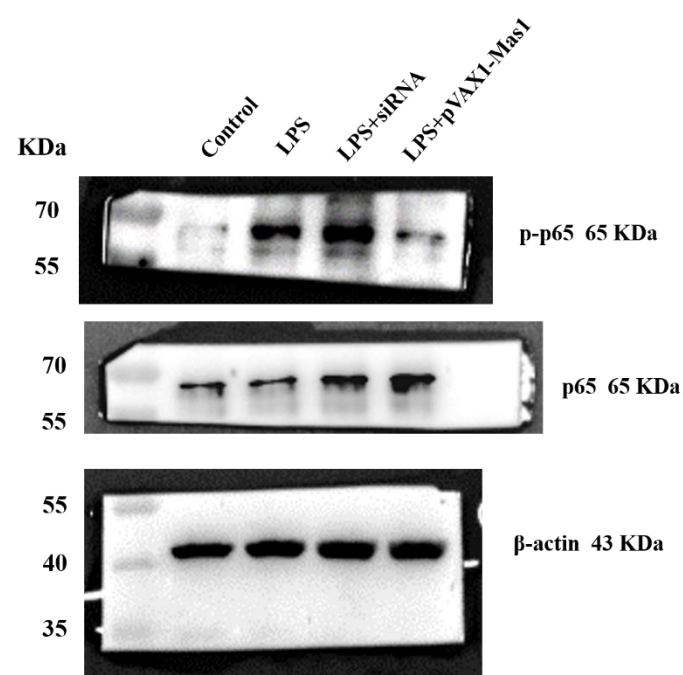

Figure 7A

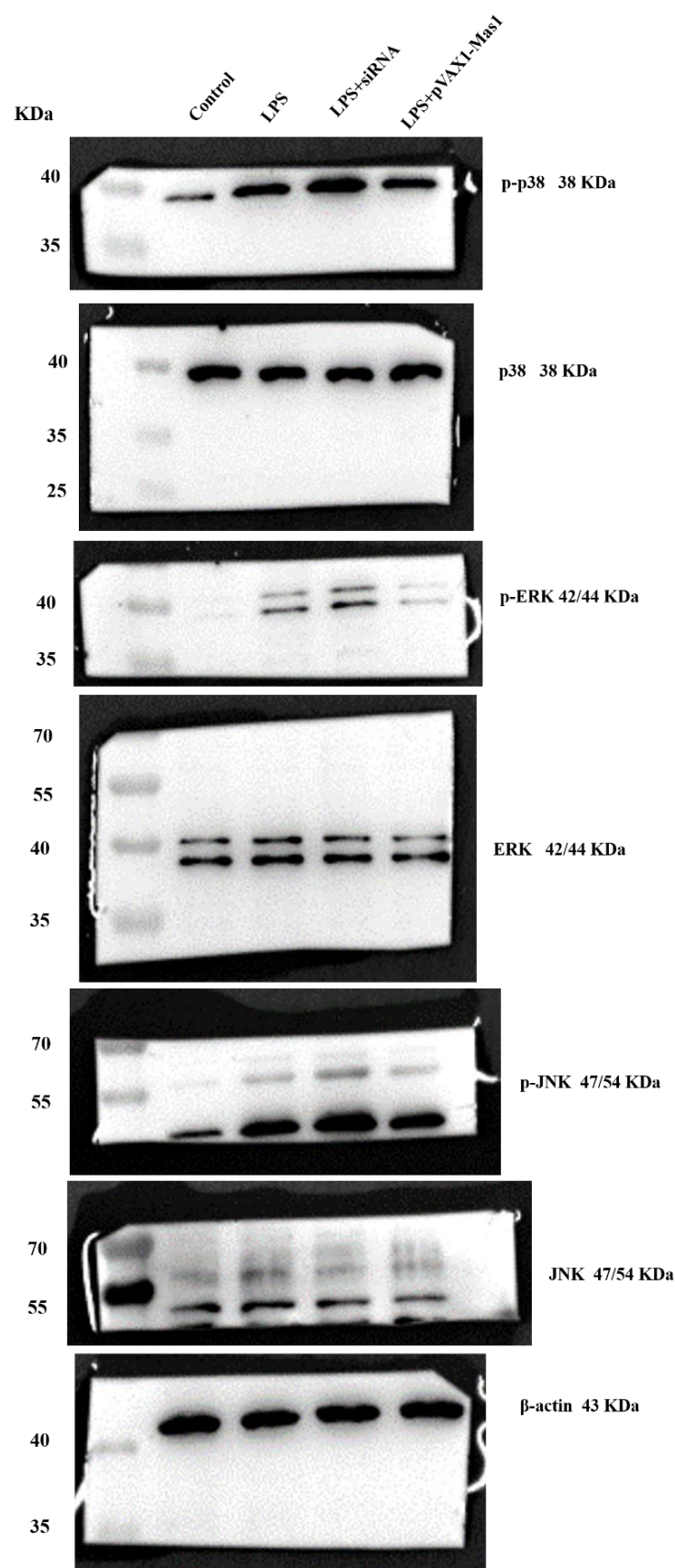

Figure 8A

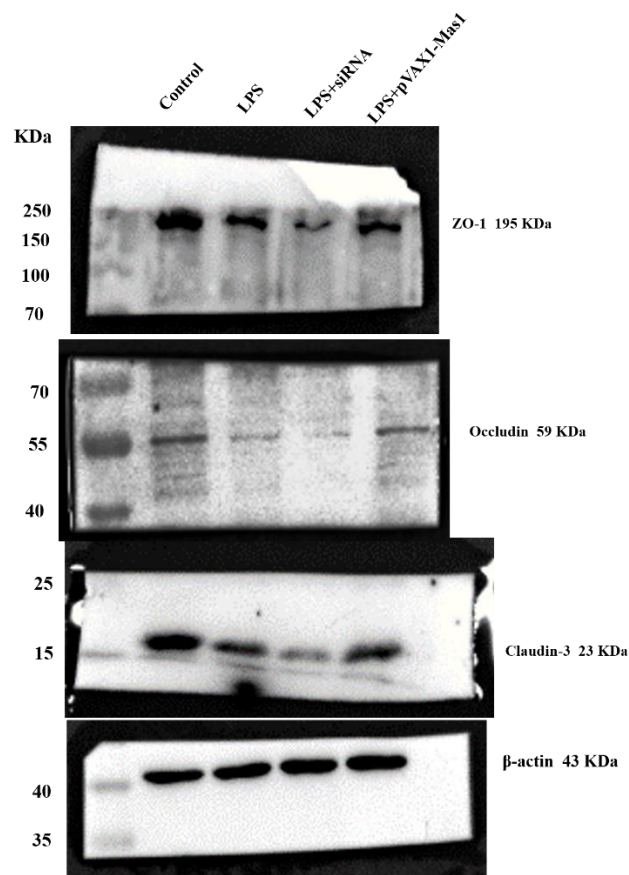

Supplement: Supplementary file 2 [file Image_1.PDF]
